# Comprehensive Analysis of Differentially Expressed Profiles of mRNA N6-Methyladenosine in Colorectal Cancer

**DOI:** 10.3389/fcell.2021.760912

**Published:** 2022-01-07

**Authors:** Na Li, Qin Guo, Qiao Zhang, Bai-Jun Chen, Xiao-An Li, Yan Zhou

**Affiliations:** ^1^ Department Of Gastroenterology, The First People’s Hospital of Aksu District, Aksu, China; ^2^ The First Affiliated Hospital of Chengdu Medical College, School of clinical medicine, Chengdu Medical College, Chengdu, China; ^3^ Mianyang Central Hospital, School of Medicine, University of Electronic Science and Technology of China, Mianyang, China

**Keywords:** colorectal cancer, MeRIP sequencing, m^6^A, RNA-binding protein, RNA-seq

## Abstract

**Aim:** To comprehensively profile the landscape of the mRNA N^6^-methyladenosine (m^6^A) modification in human colorectal cancer (CRC).

**Methods:** Methylated RNA immunoprecipitation sequencing (MeRIP-seq) was explored to compare the difference in mRNA N^6^-methyladenosine (m^6^A) methylation between CRC tissues and adjacent normal control (NC) tissue. RNA-sequencing (RNA-seq) was performed to transcribe differentially expressed mRNAs. Conjoint analysis of MeRIP-seq and RNA-seq data was conducted to predict RNA-binding proteins (RBPs).

**Results:** MeRIP-seq identified 1110 differentially m^6^A methylated sites (DMMSs) and 980 differentially m^6^A methylated genes (DMMGs) in CRC, with 50.13% of all modified genes showing unique m^6^A-modified peaks in CRC. RNA-seq showed 915 upregulated genes and 1463 downregulated genes in CRC. QRT-PCR verified the RNA-seq results by detecting the expression of some mRNAs. Conjoint analysis of MeRIP-seq and RNA-seq identified 400 differentially m^6^A methylated and expressed genes (DEGs), and pathway analysis detected that DMMGs and DEGs were closely related to cancer. After analyzing these DMMGs and DEGs through the GEPIA database, we found that the expression of B3GNT6, DKC1, SRPK1, and RIMKLB were associated with prognosis, and the expression of B3GNT6 and RIMKLB were associated with clinical stage. 17 RBPs were identified based on the DMMGs and DEGs, among which FXR1, FXR2, FMR1, IGF2BP2, IGF2BP3, and SRSF1 were obviously highly expressed in CRC, and FMR1, IGF2BP2, and IGF2BP3 were closely related to methylation, and might be involved in the development of CRC.

**Conclusion:** This study comprehensively profiled m^6^A modification of mRNAs in CRC, which revealed possible mechanisms of m^6^A-mediated gene expression regulation.

## Introduction

N^6^-methyladenosine (m^6^A) is the most abundant modification of eukaryotic mRNAs (Fustin et al., 2013). It is also widely associated with transfer RNAs, small nuclear RNAs, circular RNAs, as well as long-chain non-coding RNAs (Dominissini et al., 2012; Machnicka et al., 2013; Li and Mason, 2014; Meyer and Jaffrey, 2014; Kirchner and Ignatova, 2015; Li et al., 2017a), which participates in various biological processes in tissues and cells, including self-renewal, reproductive development, lipid metabolism, nervous system development, and immune regulation (Wang et al., 2015; Yang et al., 2016; Li et al., 2017b; Ivanova et al., 2017; Wang et al., 2018; Berry et al., 2020). RNA m^6^A modification is prevalent in plants (Kennedy and Lane, 1979), vertebrates (Desrosiers et al., 1975), viruses (Canaani et al., 1979), and bacteria (Deng et al., 2015), which has a consistently conserved sequence RRm^6^ACH [(G/A/U) (G > A) m^6^AC (U > A > C)] (Csepany et al., 1990; Narayan et al., 1994).

Three types of enzyme profiles are required for m^6^A methylation modification, including writers (m^6^A methyltransferases), erasers (m^6^A demethylases), and readers. Writers include methyltransferase 3 (METTL3, HGNC:17563), methyltransferase 14 (METTL14, HGNC:29330), Wilms tumor 1 associated protein (WTAP, HGNC: 16846), and possibly vir like m^6^A methyltransferase associated (VIRMA, HGNC:24500) and RNA binding motif protein 15 (RBM15, HGNC:14959) (Deng et al., 2018). FTO alpha-ketoglutarate dependent dioxygenase (FTO, HGNC:24678) and AlkB homolog 5 (ALKBH5, HGNC:25996) serve as m^6^A erasers, and set of YTH N6-methyladenosine RNA binding protein 1/2/3 (YTHDF1/2/3, HGNC: 15867/31675/26465), YTH domain containing 1 (YTHDC1/2, HGNC:30626/24721), and eukaryotic translation initiation factor 3 subunit A (eIF3A, HGNC:3271) play the role of m^6^A “readers” (Meyer et al., 2015; Roundtree and He, 2016; Ha et al., 2019; He et al., 2019; Liu et al., 2020a; Liu et al., 2020b; Chao et al., 2020). In addition, the function of m^6^A methylation is closely related to RNA-binding proteins (RBPs), which selectively bind to m^6^A-modified mRNAs and transport them to the mRNA degradation site to regulate mRNA metabolism (Meyer et al., 2015; He et al., 2019). These m^6^A methylation-related proteins are involved in modulating mRNA stability, shearing, transport, degradation, and translation, which regulate epigenetic changes in RNAs. Under normal physiological conditions, the dynamic balance of m^6^A methylation maintains the stable metabolism of RNA molecules. When m^6^A methylation imbalanced, the original metabolic process of RNA molecules is disrupted, resulting in epigenetic changes in RNAs.

RNA m^6^A modification is not only involved in normal metabolism in the body, but also associated with the development of different types of tumors, such as colorectal tumors (CRC), acute myeloid leukemia, non-small cell lung cancer, hepatocellular carcinoma, cervical squamous cell carcinoma, and breast cancer (He et al., 2019). A number of studies display that RNA m^6^A methylation of CRC is involved in the activity regulations of cancer Stem Cell, as well as the growth, proliferation, anti-chemotherapy and immunotherapy of tumour cells (Bai et al., 2019; Wang et al., 2020; Yang et al., 2021). Therefore, to further reveal the pathogenesis of CRC, it is worthwhile to study the modification patterns of CRC methylation.

Methylated RNA immunoprecipitation sequencing (MeRIP-seq) technology have become the main detection method for m^6^A modification (Dominissini et al., 2012). In this study, we conducted MeRIP-seq and RNA-sequencing (RNA-seq) in CRC tissues and the corresponding normal control (NC) tissues to investigate the m^6^A modification patterns in colorectal cancer, the involvement of m^6^A modification in the development of CRC, the relationship between mRNA m^6^A modification and mRNA expression, together with the predicted RBPs closely related to mRNA m^6^A methylation for a comprehensive understanding of m^6^A methylation in CRC.

## Materials and Methods

### Tissue Samples

All five specimens of patients diagnosed with CRC were obtained at the Chengdu Medical College from March 2017 to June 2018. None of these patients received radiation or chemotherapy before the specimens were collected. Clinicopathological informations of 5 CRC patients were displayed in Supplementary Table S1. Three to four specimens with diameters of 0.5–1.0 cm were collected from the center of the CRC tissues. At the same time, three to four pieces of the NC tissues of a similar size were collected 5 cm away from the edge of the cancer tissue. Informed consent was obtained from patients regarding the collection and use of these specimens. The collected specimens were frozen sectioned and stained with hemAtoxylin-eosin to confirm that the collected specimens were colon cancer (COAD) or rectal cancer (READ) tissues and corresponding NC tissues. This study had been approved by the Ethics Committee of the First Affiliated Hospital of Chengdu Medical College.

### RNA Isolation

RNA was extracted using TRIzol Reagent (Invitrogen, Carlsbad, CA, United States) according to the manufacturer’s instructions. The NEBNext rRNA Depletion Kit (New England Biolabs, Inc., Ipswich, MA, United States) was used to remove ribosomal RNA from the total RNA. The concentration and purity of RNA were detected using a NanoDrop ND-1000 spectrophotometer (Thermo, Waltham, MA, United States), and the integrity of RNA was evaluated using denaturing gel electrophoresis.

### MeRIP-Seq and RNA-Seq

MeRIP-seq and RNA-seq were provided by Cloudseq Biotech Inc. (Shanghai, China). RNA was fragmentated using RNA fragmentation reagents (Thermo, Waltham, MA, United States). A total of 5 μg of fragmented mRNA was saved as input control for RNA-Seq, while 500 μg of fragmented mRNA was used to perform m^6^A RNA immunoprecipitation with GenSeqTM m^6^A-MeRIP Kit (GenSeq, Beijing, China). Both the input samples without immunoprecipitation and the m^6^A IP samples were used to generate libraries for RNA sequencing using the NEBNext®Ultra II Directional RNA Library Prep Kit (New England Biolabs, Inc., Ipswich, MA, United States). Library quality was evaluated using a BioAnalyzer 2100 system (Agilent Technologies, Inc., Palo Alto, CA, United States). Libraries were sequenced on an Illumina HiSeq instrument 4000 with 150 bp paired-end reads. The main reagents and instruments of the research were shown in Supplementary Tables S2, S3 respectively.

### Real-Time Quantitative PCR

The cDNA primer sequences used in the study designed and synthesized by QingKe Biotechnology Co., Ltd. (Chengdu, China). The primer sequences of the target genes, β-actin and GAPDH used for RT-qPCR were displayed in Supplementary Table S4. Total RNA Extraction KIT (Solarbio, Beijing, China) was used to extract total RNA from tissues according to the manufacturer’s instructions. The concentration and purity of the isolated RNA were determined by NanoDrop ND-1000 spectrophotometer and the iScript cDNA Synthesis Kit (Bio-Rad, Hercules, CA, United States) was used for reversal. RT-qPCR was performed using SsoAdvanced Universal SYBR Green Supermix (Bio-Rad, Hercules, CA, United States). All qPCR analyses were conducted in triplicate and the average value was calculated. The expression values of genes were normalized β-actin and GAPDH, and then log2-ΔΔCt for the gene expression.

### Data Analysis

Reads were harvested from Illumina HiSeq sequencer, and were quality controlled by Q30. After 3’ adaptor-trimming and low quality reads removing by cutadapt software (v1.9.3). First, clean reads of input libraries were aligned to reference genome (UCSC HG19) by Hisat2 software (v2.0.4). After that, The MACS (v1.4.2) software compared the clean reads with the human genome to identify the methylation sites (peaks) on RNAs. Differentially m^6^A methylated sites (DMMSs, fold-change ≥ 2 and *p*-value < 0.05) were identified by diffReps (v1.55.3). Sequence motifs were identified using Homer (v3.0). Under the guidance of the Gene Transfer Format gene annotation file, gene expression was calculated by Cufflinks (v2.2.1) using FPKM values (Fragments per kilobase of exon per million fragments mapped). Fold change ≥2, *p*-value <0.05 and FPKM ≥0.1 were acted as the threshold for differentially m^6^A methylated genes (DMMGs) screening. These peaks identified by both software with overlapping mRNA exons were identified by home-made scripts. Genes of interest were visualized in the Integrative Genomics Viewer (IGV) software (v2.8.3). Screening of differentially expressed genes (DEGs) in CRC group and NC group using Cuffdiff (v2.2.1) software.

Gene ontology (GO) and Kyoto Encyclopedia of Genes and Genomes (KEGG) pathway enrichment analysis were performed by DAVID (v6.7) database. *p* < 0.05 was used as the threshold of significant enrichment.

The combined analysis of MeRIP-seq and RNA-seq screened out the genes that were differentially methylated and expressed. The differential methylation regions of CRC vs. NC were divided into 9 portions from −5 to 4 according to the methylation fold change. The binding region of RBP intersected with the differentially methylated region of the selected gene to find the RBP that can bind to the methylated region *via* RMbase (v2.0) database. [columns (Log2FC): the ratio of differentially methylated areas of CRC/NC; rows: RBPs]. The color in the figure represented the RBPs binding rate. The RBPs binding rate was calculated through the number of RBPs binding divided by the number of differential methylation regions within the interval multiplied by the number of binding information of the RBP in the database. The darker the color, the greater the binding rate, which meant that the differentially methylated regions in the range were more likely to be bound by the RBPs. The main software information used in the research was shown in Supplementary Table S5.

### Statistical Analysis

Data from three or more independent experiments were presented as mean ± standard deviation. Paired student’s *t*-tests were performed between cancer tissue samples and NC tissue samples. One-way analysis of variance was used to access the differences among five or more groups. Differences with *p* < 0.05 were considered statistically significant (*p < 0.05, **p < 0.01, ***p < 0.001).

## Results

### General Features of mRNA m^6^A Modification Patterns in CRC and NC Tissues

**FIGURE 1:**
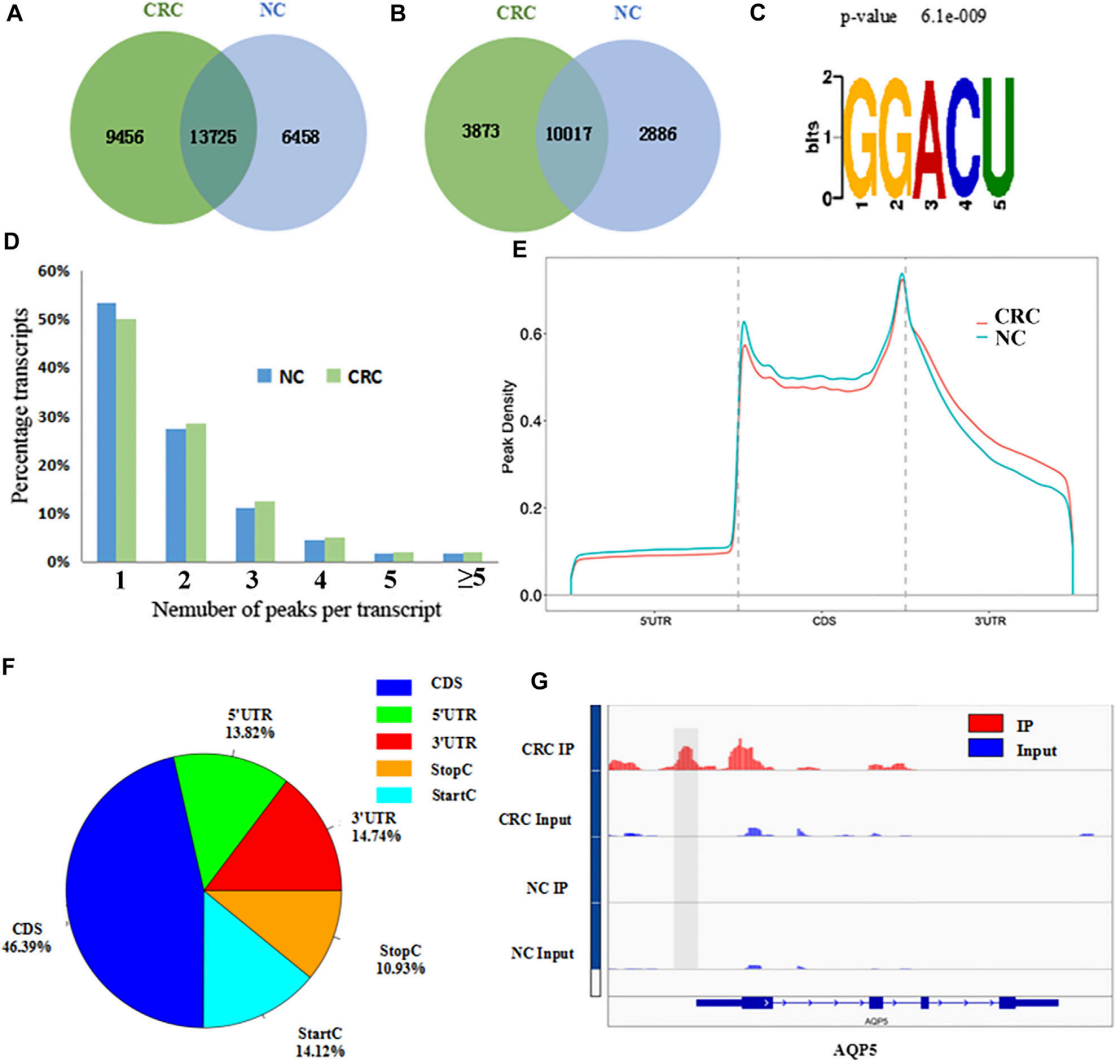
Overview of m^6^A methylation patterns in CRC tissues and NC tissues. **(A)** Venn diagram indicating the intersection of mRNA m^6^A peaks in CRC tissues and NC tissues. (CRC: colorectal cancer; NC: normal control). **(B)** The number of m^6^A peak-represented transcripts in CRC tissues and NC tissues. **(C)** Sequence motif of m^6^A peak regions. **(D)** The proportions of transcripts with different number of m^6^A peaks in CRC tissues and NC tissues. **(E)** Distribution of m^6^A peaks identified in the entire transcriptome in CRC and NC. **(F)** The percentage of m^6^A peaks in five non-overlapping segments. **(G)** Data visualization of AQP5 mRNA m^6^A modification in CRC tissues and NC tissues.

The MeRIP-seq technology was used to perform methylation sequencing on samples from CRC and NC. Reads data and quality testing were shown in Supplementary Table S6. A total of 23181 m^6^A peaks were identified in the CRC tissues, representing 13890 m^6^A-modified transcripts. In NC tissues, 20183 m^6^A peaks were identified, representing 12903 m^6^A-modified transcripts. Compared to NC tissues, CRC tissues had 9456 individual peaks and 3873 m^6^A-modified transcripts indicating a significant difference in global m^6^A modification patterns between CRC tissues and NC normal tissues (Figures 1A,B). The identified m^6^A peak was consistent with the most common m^6^A methylation motif GG/A (m^6^A) CH (Figure 1C) (Wu et al., 2019). It also confirmed the reliability of the obtained MeRIP-seq results. In addition, it was found that 50.13% of all modified genes had the unique m^6^A-modified peak in CRC tissues, and 50.33% in NC tissues. A relatively small number of m^6^A-modified genes contained two or more peaks (Figure 1D).

### Distribution of Differentially Methylated m^6^A Peaks

With respect to the m^6^A distribution patterns in the entire transcriptome, the results displayed that the m^6^A peaks were mainly enriched in the vicinity of the CDs and at the beginning of the 3’untranslated region (3′UTRs) in both groups (Figures 1E,F). IGV was used to visually analyze the differential m^6^A peak of AQP5 (HGNC:638), KISS1 (HGNC:6341) and MUC16 (HGNC:15582) in CRC and NC. The differential hypermethylated peak of AQP5 on outside of the 5′UTRs was detected. The differential hypermethylated peaks of KISS1 and MUC16 were both enriched in the CDS regions (Figure 1G, Supplementary Figure S1).

A total of 1110 DMMSs and 980 differentially m^6^A methylated genes (DMMGs) were identified in CRC tissues. Out of the 980 DMMGs, 43.78% (429/980) were significantly hypermethylated, and 56.22% (551/980) were significantly hypomethylated (Supplementary Figure S2A). The top 10 genes of m^6^A hypermethylated or hypomethylated with the highest fold-change values were shown in Table 1. There was no significant difference in the distribution pattern of hypermethylated sites. The distribution of hypomethylation sites in the 5′UTR was significantly less than the 3′ UTR and CDS regions (Supplementary Figure S2B, Supplementary Table S7). All DMMSs were mapped to chromosomes to analyze their distribution profiles. DMMSs were mostly on chromosomes 1, 2, 11, and 19 (Supplementary Figure S2C).

**TABLE 1:** The top 10 genes of m^6^A hypermethylated or hypomethylated.

Gene name	Start	End	Chrom	Regulation	Fold change
AQP5	50355278	50355320	chr12	hyper	399.5
KISS1	204159581	204159880	chr1	hyper	362
MUC16	9085801	9086000	chr19	hyper	230.66
GPR84	54756228	54756600	chr12	hyper	120.1
ARHGEF4	131673841	131674360	chr2	hyper	56.90
PDX1	28494521	28494681	chr13	hyper	47.55
NMU	56502247	56502465	chr4	hyper	41
MMP11	24115161	24115165	chr22	hyper	30.67
LRRC15	194077821	194078080	chr3	hyper	29.9
DDN	49390561	49391320	chr12	hyper	28.875
GPM6B	13835081	13835314	chrX	Hypo	382.4
GUCA2B	42619091	42619200	chr1	Hypo	180.67
CLCA4	87046081	87046432	chr1	Hypo	180.10
MYOC	171605381	171605580	chr1	Hypo	165.55
C2orf40	106682112	106682299	chr2	Hypo	164.74
XKR4	56436741	56436960	chr8	Hypo	157.5
ALK	30143861	30144260	chr2	Hypo	134.78
MYH11	15931764	15931800	chr16	Hypo	128.1
UNC13C	54306861	54307140	chr15	Hypo	118.6
SPOCK3	167656081	167656241	chr4	Hypo	116.9

### Differentially Methylated RNAs Were Involved in Important Biological Pathways

**FIGURE 2:**
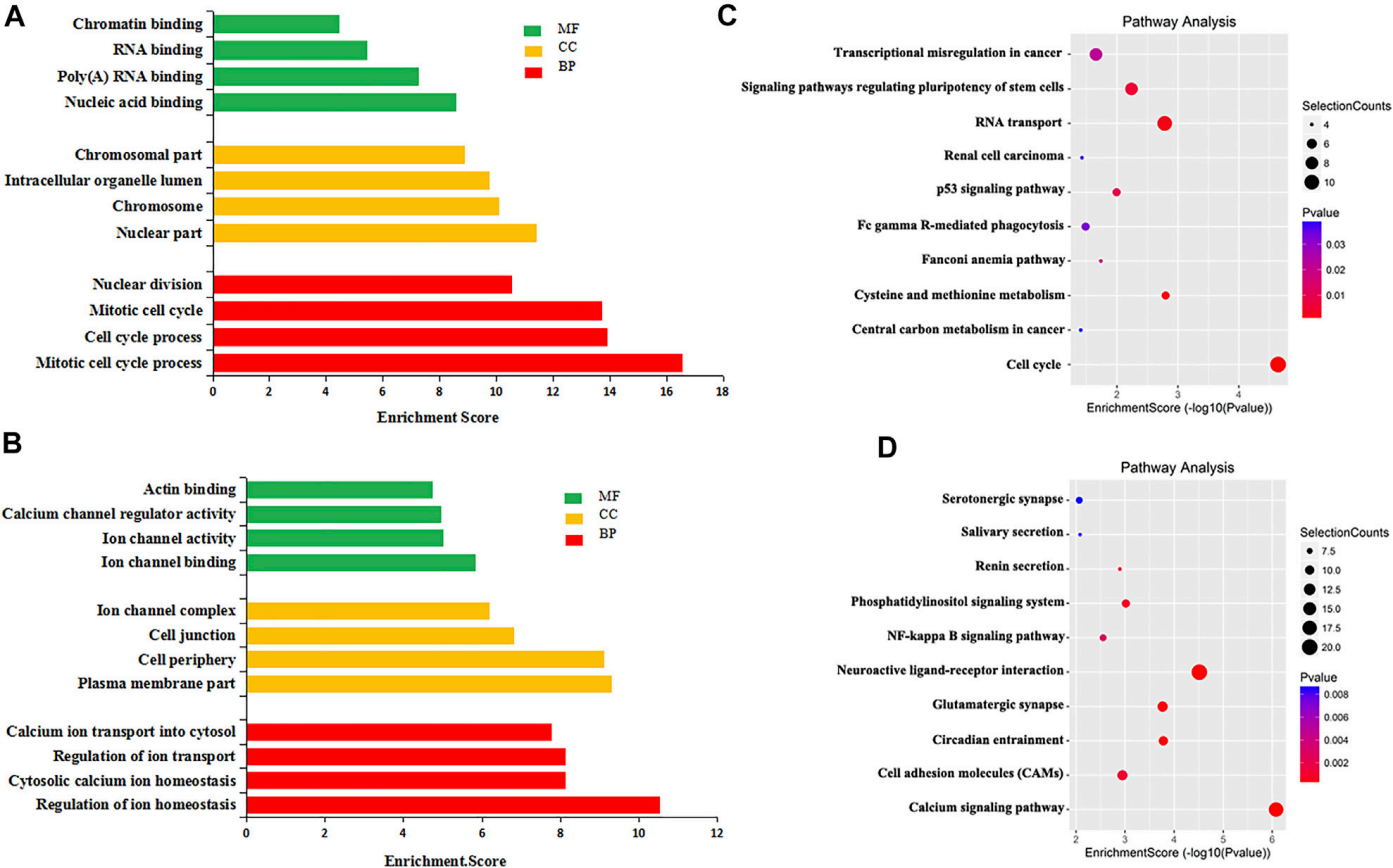
GO analysis and KEGG pathway analysis of differentially methylated mRNA. **(A)** The top 10 GO terms significantly enriched for hypermethylated genes. **(B)** The top 10 GO terms significantly enriched for hypomethylated genes. **(C)** The top 10 KEGG pathways significantly enriched for hypermethylated genes. **(D)** The top 10 KEGG pathways significantly enriched for hypomethylated genes.

To explore the biological significance of m^6^A modification in CRC tissues, GO analysis and KEGG pathway analysis were performed for DMMGs. Go analysis revealed that the hypermethylated genes were significantly enriched in the cell cycle process, intracellular organelle lumen, and nucleic acid binding (Figure 2A). The hypomethylated genes were significantly involved in the regulation of ion homeostasis, intracellular, and ion channel binding (Figure 2B). Cell cycle process, intracellular, and nucleic acid binding played an important role in tumor development (Dominissini et al., 2012).

KEGG pathway analysis revealed that hypermethylated genes were enriched in signaling pathways regulating the pluripotency of stem cells and the tumor protein p53 (TP53, HGNC:11998) signaling pathway (Figure 2C). Hypomethylated genes were enriched in the calcium signaling pathway and the nuclear factor kappa B (NF-κB, HGNC:9954) signaling pathway (Figure 2D). Stem cell pluripotency regulation, the TP53 signaling pathway, the NF-κB signaling pathway, and calcium ion regulation were closely related to the formation, metastasis, and drug resistance of multiple tumors (Ha et al., 2019; Liu et al., 2020b).

### Functional Analysis of Differentially Expressed Genes in CRC

**FIGURE 3:**
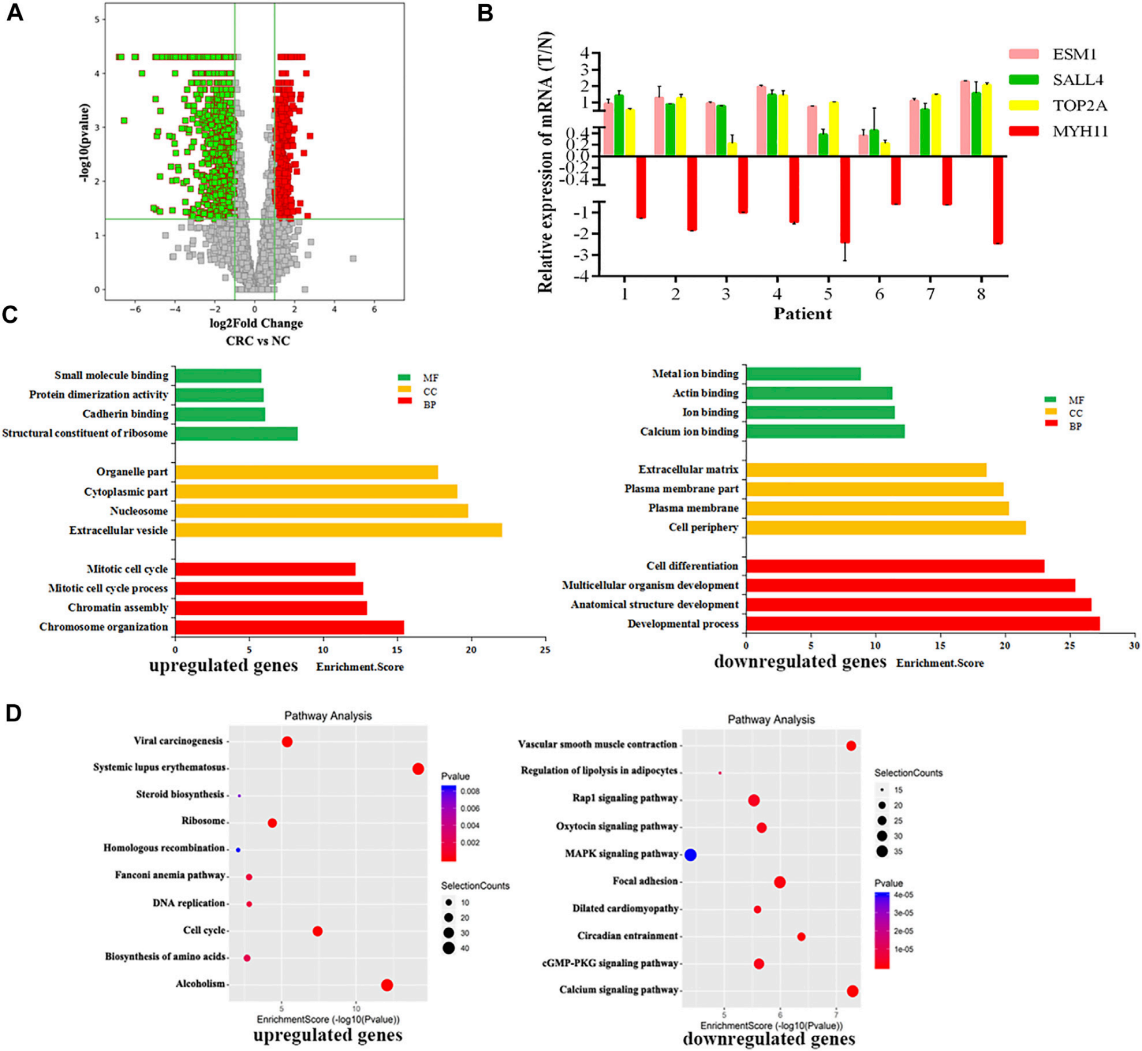
Identification of differentially expressed genes (DEGs) in CRC by RNA-seq. **(A)** The volcano plot showing the DEGs in CRC and NC groups. **(B)** The results of qRT-PCR for ESM1, SALL4, TOP2A, MYH11. **(C)** The top 10 GO terms significantily enriched for the upregulated genes on the left and downregulated genes on the right. **(D)** The top 10 GO terms significantly enriched for the upregulated genes on the left and downregulated genes on the right.

RNA-seq was used to detect DEGs (fold-change ≥ 2 and *p*-value < 0.05) in CRC and NC tissues, which showed 915 upregulated genes and 1463 downregulated genes (Figure 3A). Reads data and quality testing were shown in Supplementary Table S8. The top 10 upregulated and downregulated genes were listed in Table 2.

**TABLE 2:** The top 10 upregulated or downregulated genes.

Gene name	Chrom	Strand	Regulation	Fold change	*p*_value
CHIT1	chr1:203181954-203242769	−	Up	22.8209983269	0.0001
SMN1	chr5:69140556-70320941	+	Up	11.9389164369	0.00145
SALL4	chr20:50400580-50419059	−	Up	10.3714808994	0.0005
MAGED4B	chrX:51804922-51812368	−	Up	8.6145175405	0.01335
RP11-166B2.1	chr16:12021229-12070500	−	Up	8.28005298659	0.0126
HIST2H4A	chr1:149804220-149812765	+	Up	8.14632854479	0.02685
CCL4L2	chr17:34580901-34808104	+	Up	7.55844783378	0.0017
POU5F1B	chr8:128256881-128494384	+	Up	7.32434627799	0.0217
RBP2	chr3:139108644-139396859	−	Up	7.05252561141	0.03335
LEKR1	chr3:156543269-156763918	+	Up	6.88792770294	0.00425
RYR3	chr15:33603162-34158303	+	Down	87.3508598825	0.00005
CA7	chr16:66836777-66907159	+	Down	86.963614515	0.00105
PRIMA1	chr14:94184643-94254827	−	Down	86.2761563522	0.0488
SORCS1	chr10:108333420-10894292	−	Down	83.1393751478	0.0016
PLIN4	chr19:4472283-4517716	−	Down	79.6504164639	0.003
KIAA0408	chr6:127759550-127840500	−	Down	78.3803440206	0.00005
SYNPO2	chr4:119809995-119982402	+	Down	76.4471069275	0.00005
NAALAD2	chr11:89864682-89926062	+	Down	75.4474376718	0.01465
CHRDL1	chrX:109917083-110039286	−	Down	71.144977117	0.00005
CNN1	chr19:11649531-11661138	+	Down	68.9271280764	0.00005

Among these differentially expressed mRNAs, qRT-PCR was used to verify the expression of 4 mRNAs in 8 pairs of CRC and NC tissues. The results of qRT-PCR showed same expression trend with the RNA-Seq results. ESM1 (HGNC:3466), SALL4 (HGNC:15924), TOP2A (HGNC:11989) were upregulated, and MYH11 (HGNC:7569) was downregulated in CRC (Figure 3B).

To explore the physiological and pathological significance of DEGs in CRC, GO analysis and KEGG pathway analysis were performed. GO analysis revealed that the upregulated genes were significantly enriched in chromosome organization, extracellular organelles, and structural constituent of ribosomes, while the downregulated genes were significantly enriched in the developmental process, cell periphery, and calcium ion binding (Figure 3C). KEGG pathway analysis revealed that the upregulated genes were enriched in DNA replication and the TP53 signaling pathway, while downregulated genes were involved in the calcium signaling pathway and the MAPK signaling pathway (Figure 3D). The TP53 signaling pathway, calcium signaling pathway, and MAPK signaling pathway were important for the malignant progression of tumors (Nucera et al., 2011).

### Differentially Methylated and Expressed mRNAs Were Related to Cancer

**FIGURE 4:**
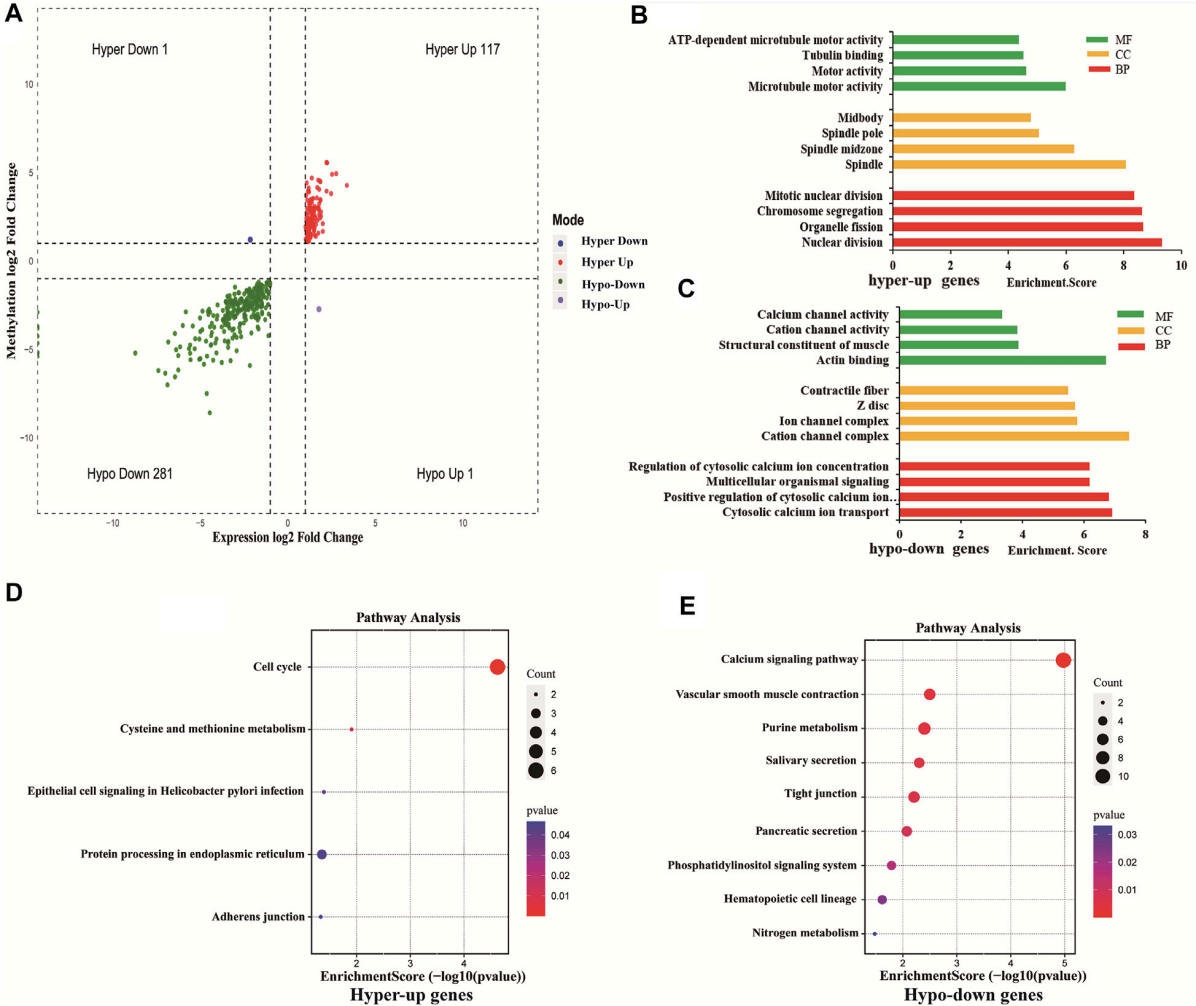
Differentially methylated and differentially expressed mRNAs were related to cancer. **(A)** Four-quadrant graph showing positive correlations between differential m^6^A methylation (fold-change ≥ 2 and *p*-value < 0.05) and differential expression (fold-change ≥ 2 and *p*-value < 0.05). **(B)** The top 10 GO terms significantly enriched for hyper-up genes. **(C)** The top 10 GO terms significantly enriched for hypo-down genes. **(D)** The top 10 KEGG pathways significantly enriched for hyper-up genes. **(E)** The top 10 KEGG pathways significantly enriched for hypo-down genes.

Using conjoint analysis of MeRIP-seq and RNA-seq data, 400 genes with differential m^6^A methylation and differential expression were identified. Among the 118 m^6^A hypermethylated genes, 117 genes had upregulated mRNA expression, and one gene had downregulated mRNA expression (Figure 4A). The top 10 hypermethylated and upregulated genes with the highest methylation fold changes were listed in Table 3. Additionally, there were 281 downregulated genes and one upregulated gene among the 282 m^6^A hypomethylated genes (Figure 4A). The top 10 hypomethylated and downregulated genes with the highest methylation fold changes were listed in Table 4. Almost all genes showed the same trend of methylation and expression. Hyper-methylation was accompanied by upregulation of mRNA expression, while hypo-methylation was accompanied by downregulation of mRNA expression. The results showed that the methylation of mRNA in CRC tissues may maintains the stability of mRNA and promotes post-transcriptional translation of mRNA.

**TABLE 3:** The top 10 hypermethylated and upregulated genes.

Gene name	Pattern	Chrom	m^6^A change		mRNA change	
			Fold change	*p*-value	Fold change	*p*-value
PDX1	Hyper-up	chr13	47.55102041	0.0000000198	4.6292228006	0.00265
NMU	Hyper-up	chr4	47.00000000	0.0000000113	4.74530230915	0.04955
MMP11	Hyper-up	chr22	30.66964286	0.0000000347	6.79451229905	0.003
LRRC15	Hyper-up	chr3	29.90000000	0.0000000235	5.77423570595	0.001
TOP2A	Hyper-up	chr17	25.86983289	0.00000063	2.5864799356	0.00475
CGREF1	Hyper-up	chr2	23.75308642	0.0000000547	3.33077984695	0.02895
ESM1	Hyper-up	chr5	22.90828139	0.000000329	2.73663999373	0.03005
MEX3A	Hyper-up	chr1	22.28901734	0.00000000349	3.63659937391	0.0016
KIF18B	Hyper-up	chr17	21.33333333	0.0000000449	2.11685140333	0.0183
SALL4	Hyper-up	chr20	19.38391225	0.000000154	10.3714808994	0.0005

**TABLE 4:** The top 10 hypomethylated and downregulated genes.

Gene name	Pattern	Chrom	m^6^A change		mRNA change	
			Fold change	*p*-value	Fold change	*p*-value
CLCA4	Hypo-down	chr1	180.1046	0.0000000814	24.8346067996	0.0015
MYH11	Hypo-down	chr16	128.1117	0.000000514	117.451391867	0.00005
CA7	Hypo-down	chr16	93.60542	0.0000000588	86.963614515	0.00105
SCN7A	Hypo-down	chr2	81.32323	0.0000000568	127.986692266	0.00005
NOS1	Hypo-down	chr12	73.25000	0.000000131	168.135435199	0.0398
SYNPO2	Hypo-down	chr4	71.57543	0.000000073	76.4471069275	0.00005
RTN1	Hypo-down	chr14	60.00000	0.00000000546	4.48444672012	0.0129
ZBTB16	Hypo-down	chr11	57.16631	0.0000000736	27.6548382729	0.0001
KIAA1683	Hypo-down	chr19	53.30000	0.00000000763	13.6916081176	0.0084
LIMS2	Hypo-down	chr2	49.80000	0.0000000334	9.63683898271	0.00005

To explore the role of differentially methylated and expressed mRNAs in the biological process of CRC, GO analysis and KEGG pathway were performed on hypermethylated-upregulated (hyper-up) genes and hypomethylated-downregulated (hypo-down) genes.

GO analysis indicated that hyper-up genes were mainly involved in the processes of microtubule motor activity, spindle and nuclear division (Figure 4B). The hypo-down genes were mainly involved in actin binding, cation channel complex, cytosolic calcium ion transport (Figure 4C). KEGG pathway analysis showed that hyper-up genes were enriched in the cell cycle and protein processing endoplasmic reticulum pathways (Figure 4D). Hypo-down genes were mainly enriched in calcium signaling pathway, vascular smooth muscle constraction and purine metabolism pathways (Figure 4E).

### Correlation Between m^6^A-Regulated Gene Expression and Clinical Parameters

**FIGURE 5:**
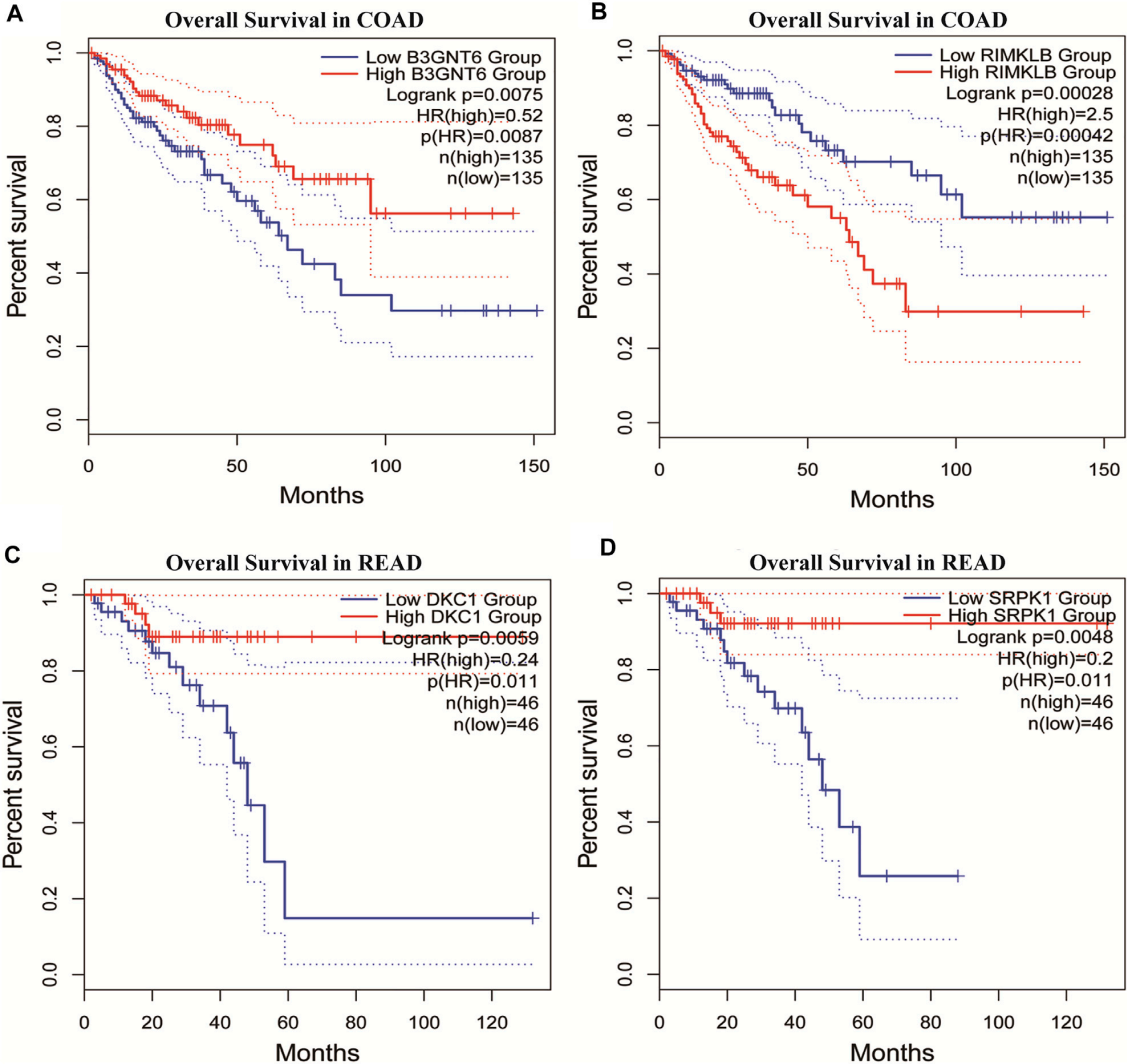
Survival analysis of differentially methylated and differentially expressed genes. **(A,B)** Survival analysis of B3GNT6/RIMKLB in COAD. **(C,D)** Survival analysis of DKC1/SRPK1 in READ. (COAD: colon cancer; READ: rectal cancer).

**FIGURE 6:**
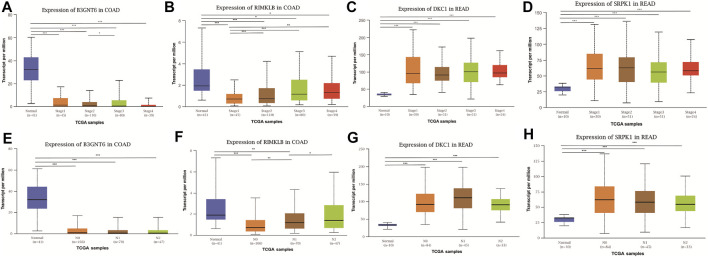
Relationship between the expression of differentially methylated and expressed genes with CRC staging and lymphatic metastasis. Expression of B3GNT6 **(A,E)**, RIMKLB **(B,F)**, DKC1 **(C,G)**, SRPK1 **(D,H)** in normal tissues or tumour tissues with different clinical staging and lymphatic metastasis. (*p < 0.05, **p < 0.01, ***p < 0.001).

To assess the clinical significance of m^6^A-regulated gene, UALCAN and GEPIA databases were explored. These databases were used to investigate the relationship between the expression of 400 differentially methylated-expressed genes with tumor staging, lymphatic metastasis, and survival period. B3GNT6 (HGNC:24141), RIMKLB (HGNC:29228) in COAD, and DKC1 (HGNC:2890), SRPK1 (HGNC:11305) in READ were analyzed. Survival analysis showed that patients with high B3GNT6, DKC1, SRPK1, and low RIMKLB expression had a longer overall survival period (Figure 5). Compared with NC, the expressions of B3GNT6, RIMKLB, DKC1, and SRPK1 in COAD or READ were significantly different, which consistented with our sequencing datas. There was a significant difference in the expression of B3GNT6 in COAD stage 2 and stage 3. The expression of RIMKLB was significantly lower in COAD stage 1 than stage 2, 3, and 4. Meawhile, the expression of RIMKLB in COAD N1 stage was different from that of N0 and N2, respectively (Figure 6).

### Conjoint Analysis of RBPs Based on Differentially Methylated and Differentially Expressed mRNA

**FIGURE 7:**
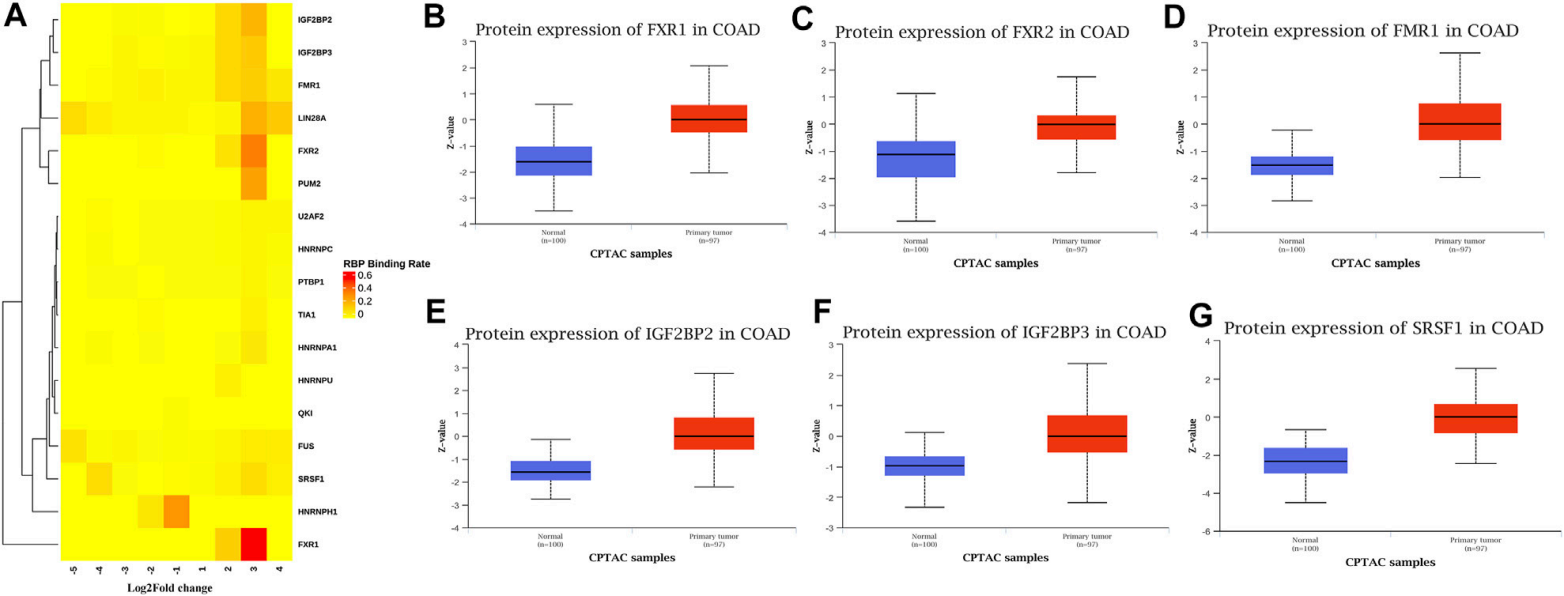
RNA binding protein based on differential methylation and expression gene prediction. **(A)** The heatmap showing 17 RBPs of differentially methylated and expressed genes (columns: the ratio of differentially methylated areas of CRC/NC; rows: RBPs). **(B–G)** Expression of FXR1/FXR2/FMR1/IGF2BP2/IGF2BP3/SRSF1 in normal tissues and primary COAD tissues based on CPTAC database. (Z-values represent standard deviations from the median across samples for COAD).

Many proteins encoded by m^6^A methylated genes played an important role in CRC tissues. The biological functions of RNAs modified by m^6^A methylation were closely related to RBPs. In order to further studied the RBPs related to m^6^A modification in the occurrence and development of colon cancer, 400 different m^6^A methylation modified and expressed genes were screened out to predict the RBPs through the RMBase database. According to the ratio of potential RBPs bound to m^6^A sites in all differentially methylated and differentially expressed mRNAs. The overview of the RBPs was presented as a heatmap (Figure 7A). In order to further verify the expression level of RBPs in colorectal cancer, we searched the protein expression in 100 NC tissues and 97 COAD tissues through the CPTAC database. Consistent with our conclusion, FXR1 (HGNC:4023), FXR2 (HGNC:4024), FMR1 (HGNC:3775), IGF2BP2 (HGNC:28867), IGF2BP3 (HGNC:28868), and SRSF1 (HGNC:10780) were obviously highly expressed in COAD (Figures 7B–G).

**FIGURE 8:**
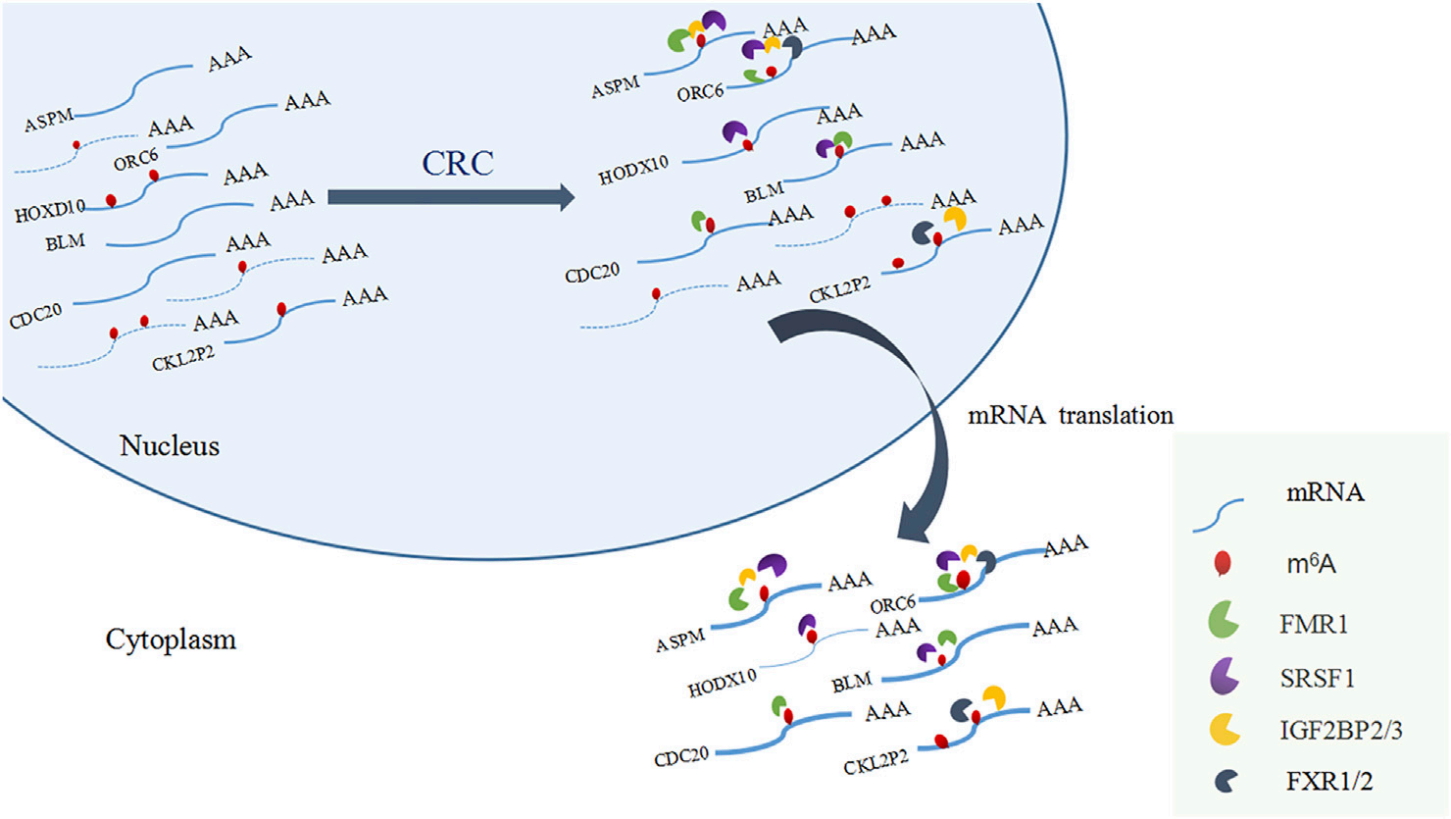
A diagram showing the pattern of m^6^A methylation regulation in CRC.

Among the 17 RBPs, FXR1/2, FMR1, PUM2 (HGNC:14958), LIN28A (HGNC:15986), IGF2BP2/3, HNRNPH1 (HGNC:5041), and SRSF1 were closely related to mRNA m^6^A methylation in CRC. FXR1/2, FMR1, PUM2, LIN28A, and IGF2BP2/3 were involved in the process of hyper-methylation. HNRNPH1 and SRSF1 were involved in the process of hypo-methylation. Specifically, FXR1/2, FMR1, IGF2BP2/3, and SRSF1 were closely related to the occurrence and malignant progress of CRC. The corresponding mRNAs of FMR1, IGF2BP2, and SRSF1 were presented in Table 5, and a diagram showing the pattern of m^6^A methylation regulation in CRC was shown in Figure 8. FXR1/2, FMR1, IGF2BP2/3, and SRSF1 were RBPs that shuttled between the nucleus and cytoplasm, which could increase the stability of targeted mRNA translation. In this study, we detected many hypermethylated and highly-expressed mRNAs related to these RBPs, which were closely related to the occurrence and malignant progression of CRC. For example, HOXD10 (HGNC:5133) was a major factor that negatively regulates tumor metastasis, and the expression of the HODX10 gene in the CRC tissue with lymphatic metastasis was lower than that in tissues without lymphatic metastasis (Wang et al., 2016). ORC6 (HGNC:17151) was related to the survival outcome of CRC (Hu et al., 2019). CDC20 (HGNC:1723), a cell cycle regulator, can bind to and activate the APC (HGNC:583) complex and participate in the development of CRC (Yu, 2007). Overexpression of the BLM (HGNC:1058) gene increased the risk of CRC (Laitman et al., 2016). Patients with a high expression of ASPM (HGNC:19048) were at more advanced clinical stages and were more prone to lymph node metastasis (An et al., 2017).

**TABLE 5:** The corresponding mRNA of FXR1/2, FMR1, IGF2BP2/3, and SRSF1.

RBPs	Gene name	m^6^A methylation		mRNA expression	
		Regulation	Fold change	Regulation	Fold change
FXR1/2	MKI67	Hyper	7.4522906793049	Up	2.56937951154
	ORC6	Hyper	11.6724053123463	Up	2.73181540363000
	CKAP2L	Hyper	8.04743083003953	Up	3.20863570191
	CDCA5	Hyper	11.0157243225159	Up	2.43842567729
	FAM111B	Hyper	7.30844293593551	Up	3.42541788293
FMR1	CKS1B	Hyper	3.3050000000000	Up	2.81540017795
	ORC6	Hyper	11.6724053123463	Up	2.73181540363000
	ASPM	Hyper	8.44098062953995	Up	2.88627747232
	MKI67	Hyper	7.26409785932722	Up	7.26409785932722
	BLM	Hyper	4.78063900810682	Up	2.08548075649
	C12orf66	Hyper	3.09634551495017	Up	2.17032334772
	CDC20	Hyper	13.0128205128205	Up	2.06166510261
	CDCA5	Hyper	11.0157243225159	Up	2.43842567729
	CENPF	Hyper	4.68294787711426	Up	2.38019537501
	FAM111B	Hyper	7.30844293593551	Up	3.42541788293
	FOXRED2	Hyper	2.94822518652498	Up	3.42541788293
	BCL2	Hypo	3.09007580214702	Down	4.6371230167
	FAM129A	Hypo	9.74852346907056	Down	16.6179440532
	AMOTL1	Hypo	7.14270687237027	Down	7.29733659166
IGF2BP2/3	ASPM	Hyper	8.44098062953995	Up	2.88627747232
	CCDC34	Hyper	5.37121212121212	Up	3.01910304853
	CDCA8	Hyper	7.90205607476635	Up	2.43293874927
	CENPF	Hyper	4.68294787711426	Up	2.38019537501
	CKAP2L	Hyper	8.04743083003953	Up	3.20863570191
	FAM111B	Hyper	7.84468664850136	Up	3.42541788293
	FOXRED2	Hyper	2.94822518652498	Up	2.40253924945
	MKI67	Hyper	7.26409785932722	Up	2.56937951154
	ORC6	Hyper	11.6724053123463	Up	2.73181540363
	ZNF485	Hyper	2.82411574366794	Up	2.55095950999
	JAM3	Hypo	5.26688311688312	Down	11.2129967914
	PCDH9	Hypo	9.03256936067551	Down	11.553835867
SRSF1	ASPM	Hyper	8.44098062953995	Up	2.88627747232
	C21orf59	Hyper	2.29333050127443	Up	2.2723105229
	BLM	Hyper	4.78063900810682	Up	2.08548075649
	TOP2A	Hyper	25.8698328935796	Up	2.5864799356
	FAM111B	Hyper	7.84468664850136	Up	3.42541788293
	ORC6	Hyper	11.6724053123463	Up	2.73181540363
	KIF14	Hyper	10.8000000000000	Up	2.15863601584
	ZNF215	Hyper	2.93900966183575	Up	2.21465197195
	MKI67	Hyper	7.45229067930490	Up	2.56937951154
	ONECUT2	Hyper	14.0484429065744	Up	5.54435640317
	MYSM1	Hyper	2.30389329488104	Down	4.4179589078
	NBEA	Hypo	8.50967741935484	Down	16.252840792
	PKNOX2	Hypo	7.03816793893130	Down	8.44290803773
	PLEKHH2	Hypo	2.60784260219396	Down	3.01581932365
	HOXD10	Hypo	19.3780487804878	Down	6.34627483195
	YJEFN3	Hypo	2.78021978021978	Down	8.10099995787

## Discussion

N^6^-methyladenosine methylation is the most abundant modification in eukaryotic mRNAs. Compared to the normal physiological process, there were cases of both hyper- and hypo-methylations in m^6^A methylation in tumors, and they both affected the development of tumors by regulating oncogene expression, cancer cell growth, survival, and invasion. METTL14 inhibited hematopoietic stem cell/progenitor cell differentiation and promotes leukemia by enhancing the m^6^A modification of MYB (HGNC:7545)/MYC (HGNC:7553) (Weng et al., 2018). YTHDF1 augmented the translation of EIF3C (HGNC:3279) in an m^6^A-dependent manner by binding to m^6^A-modified EIF3C mRNA and concomitantly promoted the overall translational output, thereby facilitating tumorigenesis and metastasis of ovarian cancer (Liu et al., 2020a). M^6^A demethylase ALKBH5 affected the proliferation and invasion of lung adenocarcinoma cells by downregulating m^6^A modification on FOXM1 (HGNC:3818) mRNA and promoting FOXM1 expression (Chao et al., 2020). Therefore, the study of methylation contributed to further understand the pathogenesis of tumors and provide directions for treatment.

In this study, MeRIP-seq and RNA-seq analyses were conducted in CRC tissues and corresponding NC tissues to profile m^6^A modification patterns in CRC. MeRIP-seq analysis revealed a total of 23181 m^6^A peaks in the CRC group, representing 13890 m^6^A-modified transcripts. Compared to the NC group, the CRC group had 9456 individual peaks and 3873 m^6^A-modified transcripts. A total of 1110 DMMSs and 980 DMMGs were identified in the CRC group, and 50% of the modified genes had the unique m^6^A-modified peak in CRC. DMMSs were more abundant on chromosomes 1, 2, 11, and 19, and predominantly distributed in the CDs and 3′ UTR.

To understand the relationship between m^6^A methylation and mRNA expression, conjoint analysis of MeRIP-seq and RNA-seq datas was conducted, and 400 genes with differential m^6^A methylation and differential expression were identified. There was a positive correlation between methylation and expression that hyper-methylation was found to be accompanied by upregulation of mRNA expression, and hypo-methylation was accompanied by downregulation of mRNA expression. Notably, m^6^A methylation can either reduce or enhance the stability, cleavage, and transport of target mRNAs (Taketo et al., 2017; Lin et al., 2019).

GO and KEGG analysis showed that the differentially methylated and expressed genes mainly enriched in Spindle and Nuclear division, calcium signaling pathway, cell cycle and protein processing endoplasmic reticulum pathways etc. Parts of pathways were closely related to cancer. Previous studies showed that ASPM was highly expressed in hepatocellular carcinoma (HCC), and the high expression of ASPM was correlated with poor HCC prognosis. Mechanism studies showed that METTL3 promoted HCC cells growth and metastasis *via* m^6^A modification of ASPM (Wang et al., 2021). Mettl14-mediated m^6^A played a critical role in liver regeneration by regulating the expression of polypeptide-processing proteins and maintaining endoplasmic reticulum homeostasis (Cao et al., 2021). The prediction of these pathways provided clues for further research on the methylation mechanism of colorectal cancer.

The regulation of m^6^A methylation was mediated by key enzymes and RBPs. RBPs prediction showed that 17 RBPs may be closely related to the differentially methylated and expressed genes in CRC. Among the 17 RBPs, FXR1, FXR2, FMR1, PUM2, IGF2BP2, IGF2BP3, and SRSF1 were closely related to mRNA m^6^A methylation in CRC. FXR1 was a new driver in the 3q26-29 amplicon and predicted poor prognosis in human cancers (Qian et al., 2015). It shuttled between the nucleus and cytoplasm, highly expressed in lung and ovarian cancer (Le Tonquese et al., 2016), and may act as a tumor promoter to increase the proliferation, migration, and invasion of colorectal cancer (Jin et al., 2016). FMR1 facilitated the nuclear export of N^6^-methyladenosine-containing mRNAs (Hsu et al., 2019). The expression of PUM2 was significantly increased in glioblastoma, and the downregulation of PUM2 can significantly inhibit the proliferation and migration of glioblastoma cells (Wang et al., 2019). METTL3 stabilized the expressions of HK2 (HGNC:4923) and SLC2A1 (HGNC:11005) in CRC through a m^6^A-IGF2BP2/3-dependent mechanism to regulate glycolytic pathways and promote colorectal cancer progression (Shen et al., 2020). Research demonstrated that IGF2BP3 bound to the mRNA of CCND1 (HGNC:1582), and reduced its mRNA stability *via* reading m6A modification in the CDs region. Overexpression of CCND1 in IGF2BP3 down-regulated cells completely rescued the inhibited percentage of S phase in cell cycle as well as cell proliferation. (Yang et al., 2020). SRSF1 was identified as a proto-oncogene that shuttled between the cytoplasm and nucleus and bound to the GGAG base sequence on mRNAs to regulate the processes of mRNA transcription, stabilization, nucleation, and translation (Long and Caceres, 2009; Das and Krainer, 2014). Long non-coding RNA AGAP2-AS1 (HGNC:48633) accelerated cell proliferation, migration, invasion and the EMT process in CRC *via* regulating the miR-4668-3p/SRSF1 axis (Li et al., 2020). The involvements of FMR1, IGF2BP2, IGF2BP3 in methylation have been preliminarily explored. However, the researchs on the mechanism of more RBPs involvement in CRC methylation deserve to be investigated further.

## Conclusion

This study established the mRNA m^6^A methylation modification map of CRC. Genes of differential m^6^A methylation and differential expression were identified and the RBPs of these genes were predicted. The possible molecular mechanism of m^6^A methylation in the development of CRC was revealed. This study provided a foundation for in-depth mechanistic analysis of m^6^A methylation in CRC.

## Data Availability

The data presented in the study are deposited in the GEO database repository, accession number GSE190388.
