# Supplementary figures and images for: Comprehensive Analysis of Differentially Expressed Profiles of mRNA N6-Methyladenosine in Colorectal Cancer
Source: Front Cell Dev Biol. 2022 Jan 7;9:760912. doi: 10.3389/fcell.2021.760912 (PMC8787460; doi:10.3389/fcell.2021.760912)

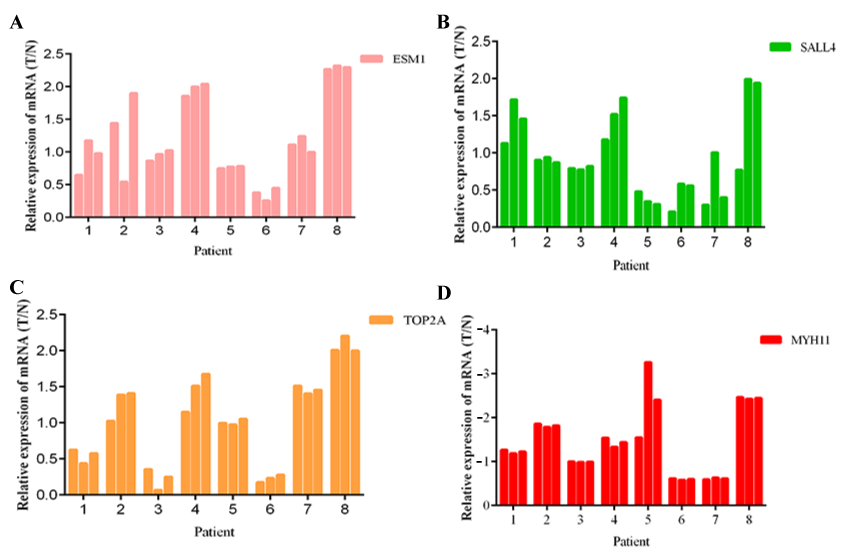

Supplement: Supplementary file 1 [file Image3.TIF]

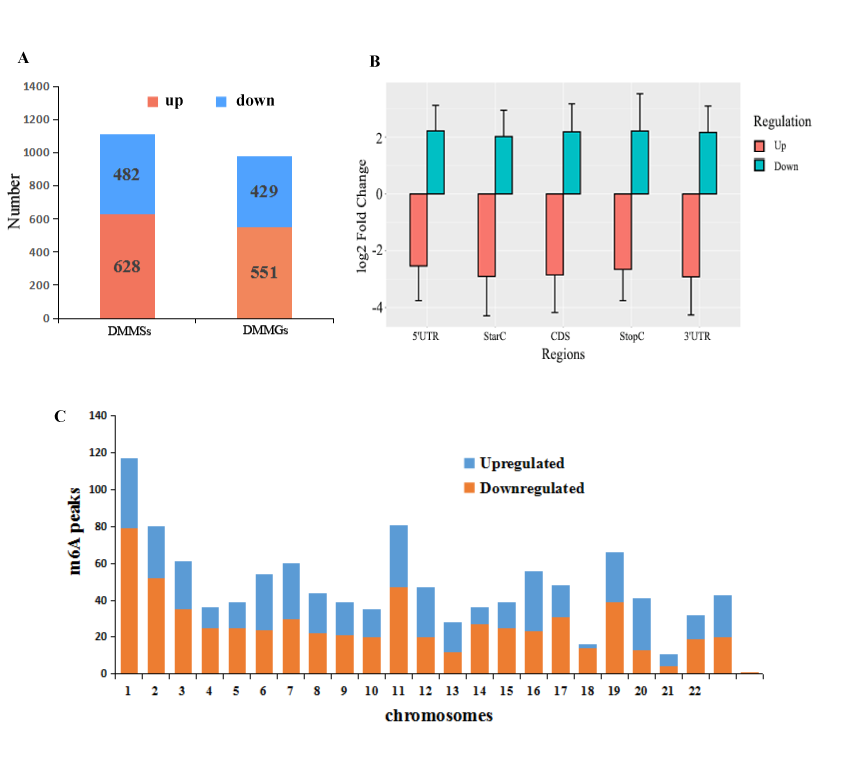

Supplement: Supplementary file 2 [file Image2.TIF]

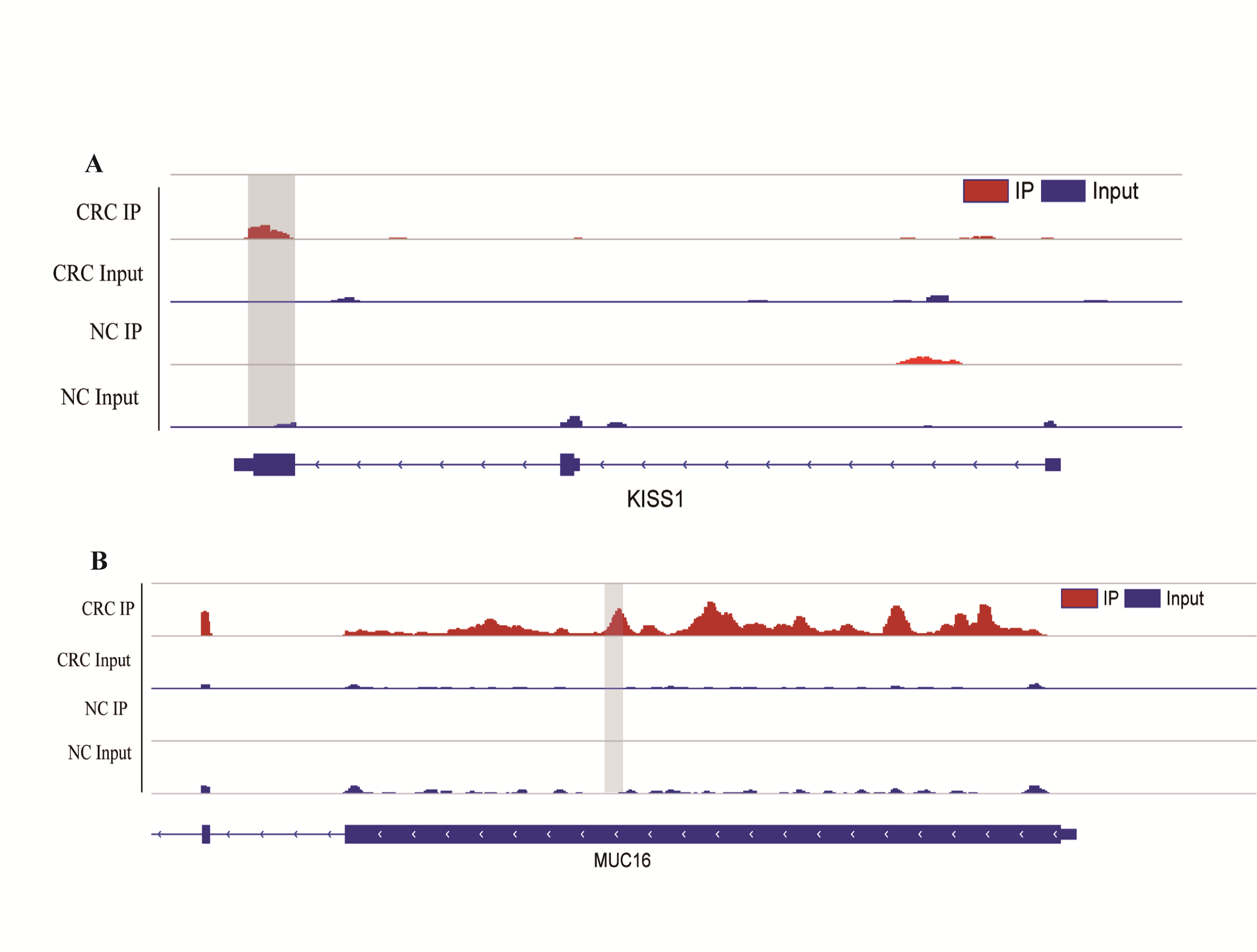

Supplement: Supplementary file 3 [file Image1.TIF]
