# Supplementary material for: Comprehensive Analysis of Differentially Expressed Profiles of mRNA N6-Methyladenosine in Colorectal Cancer
Source: Front Cell Dev Biol. 2022 Jan 7;9:760912. doi: 10.3389/fcell.2021.760912 (PMC8787460; doi:10.3389/fcell.2021.760912)
Supplement: Supplementary file 5 [file DataSheet1.docx]

**Supplementary Material**

**Supplementary Figures and Tables**

**1、Supplementary Tables**

| Supplementary Table 1: Clinical characteristics of five CRC patient |
| --- |
| **Patient ( No.) Gender Age(years) Primary location Pathological type Clinical stage** |
| 1 F 70 Rectum Adenocarcinoma T_3_N_1_M_0_  2 M 56 Ascending colon Adenocarcinoma T_4_N_0_M_0_ |
| 3 F 80 Ascending colon Adenocarcinoma T_3_N_0_M_0_  4 M 59 Rectum Adenocarcinoma T_4_N_1_M_1_  5 M 73 Ascending colon Adenocarcinoma T_1_N_0_M_0_ |

F: Female; M: Male

| Supplementary Table 2: Sources and catalog numbers of reagents used in the research |
| --- |
| **key reagents Source Catalog numbers** |
| GenSeqTM m6A-MeRIP GenSeq, Beijing, China GS-ET-001  NEBNext®Ultra II Directional  RNA Library Prep Kit New England Biolabs, Inc., Ipswich, MA, US E7760L  TRIzol Reagent Invitrogen, Carlsbad,CA， USA 15596018  NEBNext rRNA Depletion Kit Thermo, Waltham, MA, USA A39003096  Total RNA Extraction KIT Solarbio, Beijing, China R1200-50T  iScript cDNA Synthesis Kit Bio-Rad, Hercules, CA, USA 170-8891  SsoAdvanced Universal SYBR  Green Supermix Bio-Rad, Hercules, CA, USA 172-5272 |

| Supplementary Table 3: Instruments used in the research |
| --- |
| **Instrument Source RRID** |
| NanoDrop ND-1000 Thermo, Waltham, MA, USA SCR_016517  BioAnalyzer 2100 Agilent Technologies Inc., Palo Alto, CA, USA SCR_019389  Illumina HiSeq 4000 Illumina, Inc, San Diego, CA, USA SCR_020127 |

| Supplementary Table 4: QRT-PCR primer sequences used in the research | |
| --- | --- |
| **Gene name Primer (5′ -3′) Sequence Product size (bp)** | |
| MYH11    ESM1 | Forward GACAAGACCCATCGGCAAGG 88  Reverse GCTGCTCGAAGGAGTTCACC  Forward AAACTTGCTACCGCACAGTC 81  Reverse CCTCCCCATTAGAAGGCTGA  Forward GCGGAGAGGGCAAATAACTG 101  Reverse GTCCTCCTCCGAGTTGATGT  Forward GTTCTTGAGCCCCTTCACGA 142  Reverse CCAATGTAGGTGTCTGGGCG  Forward CCTTCCTGGGCATGGAGTC 189  Reverse TGATCTTCATTGTGCTGGGTG  Forward GGAGTCCACTGGCGTCTTCA 240  Reverse GTCATGAGTCCTTCCACGATACC |
|  |  |
| SALL4 |  |
| TOP2A  β-actin  GAPDH |  |

| Supplementary Table 5: Softwares used in the research |
| --- |
| **Software RRID Software link** |
| Hisat2 SCR_015530 http://ccb.jhu.edu/software/hisat2/index.shtml  MACS SCR_013291 https://pypi.org/project/MACS/1.4.2/  DiffReps SCR_010873 https://metacpan.org/dist/diffReps  IGV SCR_011793 https://software.broadinstitute.org/software/igv/download  Cutadapt SCR_011841 https://github.com/marcelm/cutadapt/  Cufflinks SCR_014597 http://cufflinks.cbcb.umd.edu/  DAVID database SCR_001881 https://david.ncifcrf.gov/  CPTAC database SCR_017135 https://cptac-data-portal.georgetown.edu/studies  UALCAN database SCR_015827 http://ualcan.path.uab.edu/  GEPIA databases SCR_018294 <http://gepia.cancer-pku.cn/>  Homer SCR_010881 <http://homer.ucsd.edu/homer/motif/>  Cuffdiff SCR_001647 http://cole-trapnell-lab.github.io/cufflinks/ |

| Supplementary Table 6: Summary of reads data and quality testing of MeRIP-seq | | | | | | |
| --- | --- | --- | --- | --- | --- | --- |
| **Sample Raw reads Clean reads MappedReads Mapped Ratio Q30（%）** | | | | | | |
| CRC1 | IP | 82256520 | 82087502 | 67562949 | 83.21% | 94.61 |
|  | Input | 75059650 | 75037608 | 69693670 | 92.88% | 93.75 |
| CRC2 | IP | 87627170 | 87511266 | 78358181 | 89.54% | 94.11 |
|  | Input | 85057806 | 85036922 | 73412640 | 86.33% | 93.58 |
| CRC3 | IP | 81711732 | 81585714 | 69639248 | 85.36% | 94.78 |
|  | Input | 70803886 | 70777414 | 65596825 | 92.68% | 93.14 |
| CRC4 | IP | 82572292 | 82356104 | 62374168 | 75.74% | 93.66 |
|  | Input | 69429010 | 69389894 | 61750504 | 88.99% | 93.86 |
| CRC5 | IP | 84231868 | 84058888 | 68048572 | 80.95% | 93.10 |
|  | Input | 83559328 | 83536956 | 67849869 | 81.22% | 93.91 |
| NC1 | IP | 82412580 | 82215222 | 61568309 | 74.89% | 94.18 |
|  | Input | 95860624 | 95763484 | 81019133 | 84.60% | 93.58 |
| NC2 | IP | 82334070 | 82152210 | 65145948 | 79.30% | 94.03 |
|  | Input | 83659056 | 82891206 | 57375416 | 69.22% | 86.15 |
| NC3 | IP | 82762068 | 82515736 | 59031716 | 71.54% | 93.11 |
|  | Input | 69916896 | 69875050 | 61679786 | 88.27% | 93.87 |
| NC4 | IP | 84047652 | 83718218 | 58051309 | 69.34% | 92.29 |
|  | Input | 78250592 | 78167884 | 60798927 | 77.78% | 93.11 |
| NC5 | IP | 83882646 | 83272544 | 45300892 | 54.40% | 90.15 |
|  | Input | 80231304 | 80008376 | 54996057 | 68.74% | 92.06 |

| Supplementary Table 7: Statistical Analysis of the Distribution Patterns of Methylation Up-regulation and Methylation Down-regulation |
| --- |
| **groupA groupB wilcox.test.P** |
| up_3UTR up_5UTR 0.56845112  up_3UTR up_CDS 0.80789104  up_3UTR up_startC 0.31035671  up_3UTR up_stopC 0.77596672  up_5UTR up_CDS 0.65207545  up_5UTR up_startC 0.14631886  up_5UTR up_stopC 0.39223624  up_CDS up_startC 0.09078495  up_CDS up_stopC 0.47045358  up_startC up_stopC 0.52385838  down_3UTR down_5UTR 0.04465217  down_3UTR down_CDS 0.73411258  down_3UTR down_startC 0.90288897  down_3UTR down_stopC 0.477231  down_5UTR down_CDS 0.03574013  down_5UTR down_startC 0.09211122  down_5UTR down_stopC 0.2965787  down_CDS down_startC 0.86936697  down_CDS down_stopC 0.59646744  down_startC down_stopC 0.5086349 |

| Supplementary Table 8: Summary of reads data and quality testing of RNA-seq | | | | | | | |
| --- | --- | --- | --- | --- | --- | --- | --- |
| **Sample** | | **Rew reads** | **Clean reads** | **Clean ration**  **(%)** | **Mapped**  **reads** | **Mapped Ration****（%）** | **Q30**  **(%)** |
| CRC1 | 75059650 | | 75037608 | 99.97 | 68149306 | 90.82 | 93.75 |
| CRC2 | 85057806 | | 85036922 | 99.98 | 72564594 | 85.33 | 94.11 |
| CRC3 | 70803886 | | 70777414 | 99.96 | 64071154 | 90.52 | 93.14 |
| CRC4 | 69429010 | | 69389894 | 99.94 | 61024952 | 87.95 | 93.86 |
| CRC5 | 83559328 | | 83536956 | 99.97 | 66495986 | 79.60 | 93.91 |
| NC1 | 95860624 | | 95763484 | 99.90 | 80466640 | 84.03 | 93.58 |
| NC2 | 83659056 | | 82891206 | 99.08 | 58532912 | 70.61 | 86.15 |
| NC3 | 69916896 | | 69875050 | 99.94 | 60701558 | 86.87 | 93.87 |
| NC4 | 78250592 | | 78167884 | 99.89 | 60207990 | 77.02 | 93.11 |
| NC5 | 80231304 | | 80008376 | 99.72 | 55234294 | 69.04 | 92.06 |

**2. Supplementary Figures**

**Supplementary Figure 1.** IGV figures to visually display the differential m^6^A peak of KISS1 (A) and MUC16 (B) in CRC and NC.

**Supplementary Figure 2.** Distribution mode of differentially methylated m6A sites. (A) Quantitative statistics of DMMSs and DMMGs. (B) The proportion of fold enrichment of DMMSs in five segments in CRC. (C) The distributions of DMMSs in human chromosomes.
